# Supplementary material for: Dopamine and calcium dynamics in the nucleus accumbens core during food seeking
Source: bioRxiv. 2025 Mar 15:2025.03.11.642710. Preprint. [Version 2] doi: 10.1101/2025.03.11.642710 (PMC11952458; doi:10.1101/2025.03.11.642710)
Supplement: Supplement 1 [file NIHPP2025.03.11.642710v2-supplement-1.pdf]

bioRxiv preprint doi: https://doi.org/10.1101/2025.03.11.642710; this version posted March 15, 2025. The copyright holder for this preprint (which was not certified by peer review) is the author/funder, who has granted bioRxiv a license to display the preprint in perpetuity. It is made available under aCC-BY 4.0 International license.

Supplementary Information (Figures S1 and S2, Table S1)

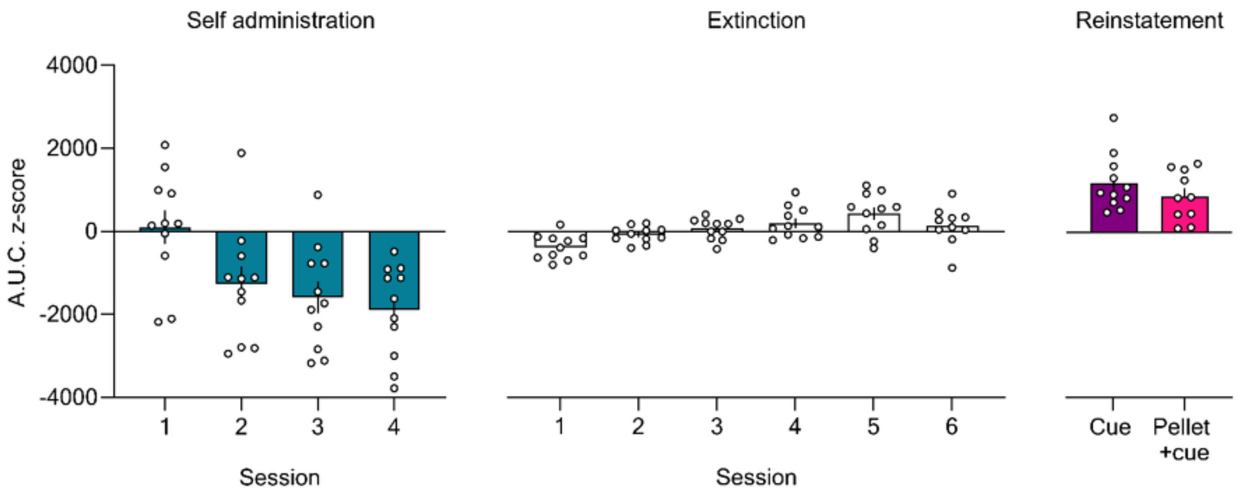

Figure S1. Area under the curve (AUC) for GCaMP traces across all recorded sessions. The time period analyzed corresponds to that shown in Fig. 2, i.e., 0 to 3 s after the lever press. Bars show mean (±SEM) AUC while dots indicate individual rats.

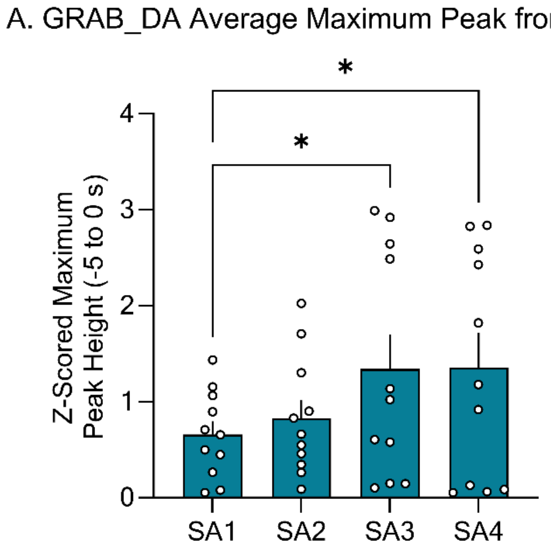

Figure S2. Z-scored maximum peak height for GRAB_DA traces across all SA sessions. The time period analyzed corresponds to the 5 sec prior to the lever press. Data were analyzed with a one-way ANOVA. Bars show mean (±SEM) Maximum Peak while dots indicate individual rats.

bioRxiv preprint doi: https://doi.org/10.1101/2025.03.11.642710; this version posted March 15, 2025. The copyright holder for this preprint (which was not certified by peer review) is the author/funder, who has granted bioRxiv a license to display the preprint in perpetuity. It is made available under aCC-BY 4.0 International license.

Table S1. Statistical output for behavioral data in Figure 1

Expt phase	Measure	Fixed effects	F-value	P-value	Significant?	Figure
SA4, Ext6, Cue, Pellet+cue	Lever presses (n = 11, 6M/5F)	Session x Lever				1E
		Session	$F_{3,30}$=68.67	<0.0001	****	
		Lever	$F_{1,10}$=88.86	<0.0001	****	
		Session x Lever	$F_{3,29}$=23.85	<0.0001	****	
		Holm-Šídák†				
		Inactive				
		SA4 vs. Ext6	t_{59}=3.779	0.0022	**	
		SA4 vs. Cue	t_{59}=2.995	0.0159	*	
		SA4 vs. Pellet+cue	t_{59}=3.542	0.0039	**	
		Ext6 vs. Cue	t_{59}=0.6811	0.8739	n.s.	
		Ext6 vs. Pellet+cue	t_{59}=0.2376	0.8805	n.s.	
		Cue vs. Pellet+cue	t_{59}=0.4500	0.8805	n.s.	
		Active				
		SA4 vs. Ext6	t_{59}=14.77	<0.0001	****	
		SA4 vs. Cue	t_{59}=11.79	<0.0001	****	
		SA4 vs. Pellet+cue	t_{59}=12.24	<0.0001	****	
		Ext6 vs. Cue	t_{59}=2.980	0.0125	*	
		Ext6 vs. Pellet+cue	t_{59}=2.527	0.0282	*	
		Cue vs. Pellet+cue	t_{59}=0.4535	0.6518	n.s.	

†adjusted P-values reported for post hoc comparisons. M, male, F, female.

Table S2. Statistical output for bootstrapping analyses in Figure 2

Expt phase	Measure	Factors in analysis	Time 95% CI ≠ 0	Significantly different?	Figure
SA	GCaMP response to lever press, z-scored trace (n=11)	Bootstrapping		0.235 to 3.51 s	2A, left
		SA1	n.s.		
		SA4	0.375 to 6.69 s		
Extinction	GCaMP response to lever press, z-scored trace (n=11)	Bootstrapping		n.s.	2B, left
		Ext1	0.394 to 1.84 s		
		Ext6	n.s.		
Extinction/ Reinstatement	GCaMP response to lever press, z-scored trace (n=11)	Bootstrapping		n.s.	2C, left
		Ext6	n.s.		
		Cue test	-0.214 to 4.46 s		
Extinction/ Reinstatement	GCaMP response to lever press, z-scored trace (n=11)	Bootstrapping		n.s.	2D, left
		Ext6	n.s.		
		Pellet+cue test	n.s.		

bioRxiv preprint doi: https://doi.org/10.1101/2025.03.11.642710; this version posted March 15, 2025. The copyright holder for this preprint (which was not certified by peer review) is the author/funder, who has granted bioRxiv a license to display the preprint in perpetuity. It is made available under aCC-BY 4.0 International license.

Table S3. Statistical output for AUC GCaMP fiber photometry data in Figure 2

Expt phase	Measure	Comparison	T-value	P-value	Significant?	Figure
SA	AUC (n = 11)	SA1 vs. SA4	t_{10}=5.143	0.0004	***	2A, right
Extinction	AUC (n = 10)	Ext1 vs. Ext6	t_9=3.000	0.0150	*	2B, right
Extinction/ reinstatement	AUC (n = 10)	Ext6 vs. Cue	t_9=3.413	0.0077	**	2C, right
Extinction/ reinstatement	AUC (n = 9)	Ext6 vs. Pellet+cue	t_8=2.393	0.0436	*	2D, right

Table S4. Statistical output for bootstrapping analyses in Figure 3

Expt phase	Measure	Factors in analysis	Time 95% CI ≠ 0	Significantly different?	Figure
SA	GRAB_DA response to lever press, z-scored trace (n=11)	Bootstrapping		-2.01 to -1.07 s, 0.285 to 1.53 s, 1.79 to 5.23 s, 5.56 to 6.83 s, 8.55 to 10.0 s	3A, left
		SA1	-1.82 to -0.507 s, 1.79 to 3.55 s		
		SA4	-5.00 to -1.41 s, -1.13 to 1.77 s, 2.38 to 10.0 s		
Extinction	GRAB_DA response to lever press, z-scored trace (n=11)	Bootstrapping		n.s.	3B, left
		Ext1	to -2.89 s, 0.71 to 7.44 s, 7.77 to 9.75 s		
		Ext6	n.s.		
Extinction/ Reinstatement	GRAB_DA response to lever press, z-scored trace (n=11)	Bootstrapping		n.s.	3C, left
		Ext6	n.s.		
		Cue test	n.s.		
Extinction/ Reinstatement	GRAB_DA response to lever press, z-scored trace (n=11)	Bootstrapping		n.s.	3D, left
		Ext6	n.s.		
		Pellet+cue test	n.s.		

Table S5. Statistical output for AUC GRAB_DA fiber photometry data in Figure 3

Expt phase	Measure	Comparison	T-value	P-value	Significant?		Figure
SA	AUC (n = 11)	SA1 vs. SA4	t_{10}=1.970	0.0856	n.s.		3A, right
Extinction	AUC (n = 10)	Ext1 vs. Ext6	t_9=0.4708	0.6490	n.s.		3B, right
Extinction/ reinstatement	AUC (n = 10)	Ext6 vs. Cue	t_9=2.589	0.0293	*		3C, right
Extinction/ reinstatement	AUC (n = 10)	Ext6 vs. Pellet+cue	t_9=2.519	0.0359	*		3D, right

bioRxiv preprint doi: https://doi.org/10.1101/2025.03.11.642710; this version posted March 15, 2025. The copyright holder for this preprint (which was not certified by peer review) is the author/funder, who has granted bioRxiv a license to display the preprint in perpetuity. It is made available under aCC-BY 4.0 International license.

Table S6. Statistical output for Peak Height GRAB_DA fiber photometry data in Figure S2

Expt phase	Measure	Fixed effects	F-value	P-value	Significant?	Figure
SA	Peak Height (n = 11)	Session	$F_{3,30}$=4.396	0.0112	*	S2A
		Holm-Šídák†				
		SA1 vs. SA2	t_{30}=0.7093	0.4836	n.s.	
		SA1 vs. SA3	t_{30}=2.843	0.0205	*	
		SA1 vs. SA4	t_{30}=2.903	0.0205	*	
